# Effects of the Van der Waals Force on the Vibration of Typical Multi-layered Two-dimensional Nanostructures

**DOI:** 10.1038/s41598-020-57522-9

**Published:** 2020-01-20

**Authors:** Yiqing Zhang, Lifeng Wang

**Affiliations:** State Key Laboratory of Mechanics and Control of Mechanical Structures, Nanjing University of Aeronautics and Astronautics, 210016 Nanjing, China

**Keywords:** Mechanical properties, Structural properties

## Abstract

Recently, two-dimensional nanostructures have caught much attention because of their magnificent physical characteristics. The vibrational behavior of typical multi-layered two-dimensional nanostructures (TMLTNs) is extraordinary significant to TMLTN-based nanoresonantors. In this investigation, the vibrational behavior of TMLTNs, taking black phosphorus (BP), graphene and BN as examples, is studied adopting molecular dynamics (MD) simulations and the sandwich plate model (SPM). The MD results show that the fundamental resonant frequency of multi-layered BP (MLBP) and multi-layered BN (MLBN) increase obviously with the number of layers. However, the fundamental resonant frequency of a multi-layered graphene sheet (MLGS) rise slightly when the number of layers increases. This phenomenon is caused by the shear modulus in the *xz*-plane and *yz*-plane resulted by the vdW force. Hence, an SPM considering the shear modulus in the *xz*-plane and *yz*-plane caused by the vdW force is used to investigate the vibration of the TMLTN. Compared with the MD results, it is shown that the SPM can better predict the vibration of the TMLTN.

## Introduction

In recent years, two-dimensional materials have caught much attention. Graphene shows great mechanical properties, with an elastic modulus of 1 TPa^1^, and it as well displays unique electronic and optical properties that have potential applications in photonic and optoelectronic devices^2–5^. Black phosphorus (BP) and boron nitride (BN) both have a wide band gap and can thus be applied in semiconductor technology^6–13^. BP can be used as a promising nanoresonator with a high resonant frequency^14,15^. BN also has extremely good mechanical and thermal properties^16–19^.

The vdW force is especially important for two-dimensional nanostructures. In a recent study, Li and his coworkers successfully probed the van der Waals (vdW) interactions of two-dimensional heterostructures via experiments^20^. Lin and Zhao^21^ utilized the theory and simulations to explore the mechanical peeling of vdW heterostructures. Zhao^22^ presented the role of vdW force in the crossover from continuum mechanics to mesoscopic mechanics. Xu and Zheng^23^ gave a brief review on progress and perspectives of the micro- and nano-mechanics. Many scholars have focused on the vibration of multi-layered nanostructures considering the vdW interactions between layers^24,25^, and their results have shown that the vdW interaction and number of layers have no influence on the fundamental natural frequencies. Liu *et al*.^26^ explored the influence of interlayer shear on the multi-layered graphene sheet (MLGS). Understanding the influence of the vdW force on the dynamic behavior of typical multi-layered two-dimensional nanostructures (TMLTNs) is still a challenge. Different two-dimensional materials have different properties. For example, BP is a highly anisotropic material^27–29^, while graphene and BN are isotropic materials^30–32^. The vdW force of a TMLTN has a different influence on the dynamic behavior. Hence, establishing a continuum model to investigate the dynamic behavior of TMLTNs would enable thoroughly to understand the vibration of TMLTNs. However, few investigations have studied the vibration of TMLTNs with the effects of the vdW force taken into account.

In this paper, we use the molecular dynamics (MD) simulation and sandwich plate model (SPM) to study the dynamic behavior of TMLTNs considering the interlayer shear effect caused by the vdW interactions, taking multi-layered BP (MLBP), MLGS and multi-layered BN (MLBN) as examples. The different influence of interlayer shear effect caused by the vdW force on the vibration of TMLTNs is studied.

## Results and Discussions

**Figure 1:**
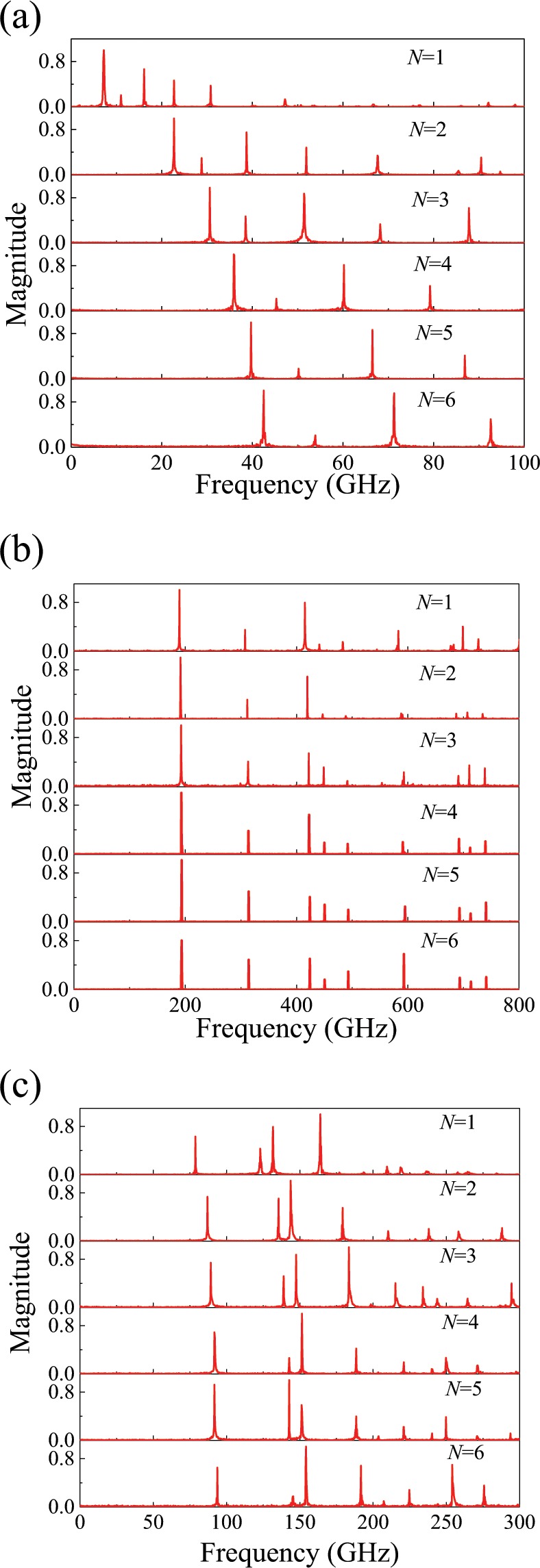
Vibration spectra of the TMLTN. (**a**) MLBP (*a* = 16.56 nm, *b* = 25.67 nm), (**b**) MLGS (*a* = 11.31 nm, *b* = 10.47 nm), and (**c**) MLBN (*a* = 11.54 nm, *b* = 10.52 nm) from one to six layers.

**Figure 2:**
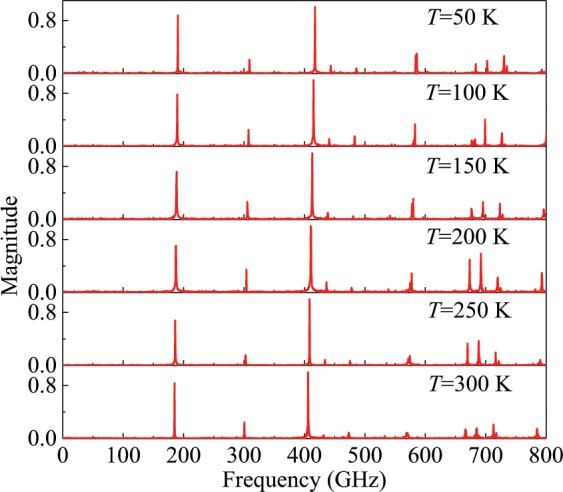
Vibration spectra of single-layered graphene sheet at different temperature.

The dynamic behavior of TMLTNs is explored. The vibration spectra of the MLGS, MLBP and MLBN obtained from MD simulations are illustrated in Fig. 1(a–c). The MD simulations are conducted in a canonical (NVT) ensemble at 100 K with a time step of 1 fs. The displacement of the atoms is recorded every 100 steps. In Fig. 1(a–c), each peak in the vibrational spectra represents one resonant frequency. As shown in Fig. 1(a–c), the resonant frequency of the MLBP and MLBN increases obviously with the number of layers. However, the resonant frequency of the MLGS exhibits little increase. Figure 2 shows vibration spectra of single-layered graphene sheet at different temperature. From Fig. 2, it can be seen that the temperature has little influence on the vibration of TMLTN.

**Figure 3:**
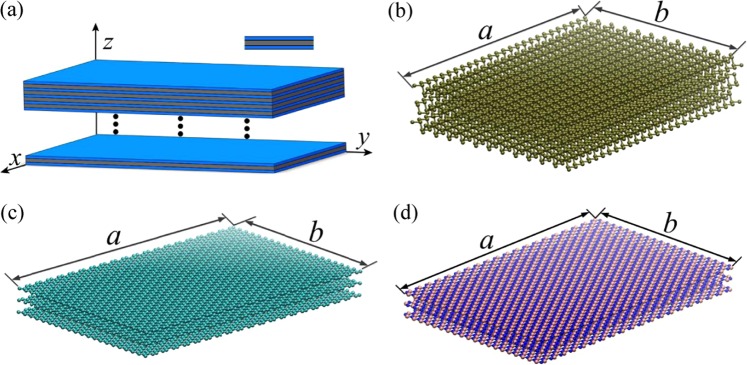
Models of the TMLTN. (**a**) The equivalent continuum model. Molecular model of (**b**) MLBP, (**c**) MLGS, (**d**) MLBN. (a is created by office 2016, URL is http://kms.nuaa.edu.cn/#zb_rjxz. (**b–d**) are created by VMD 2019, URL is https://www.ks.uiuc.edu/Research/vmd/).

**Figure 4:**
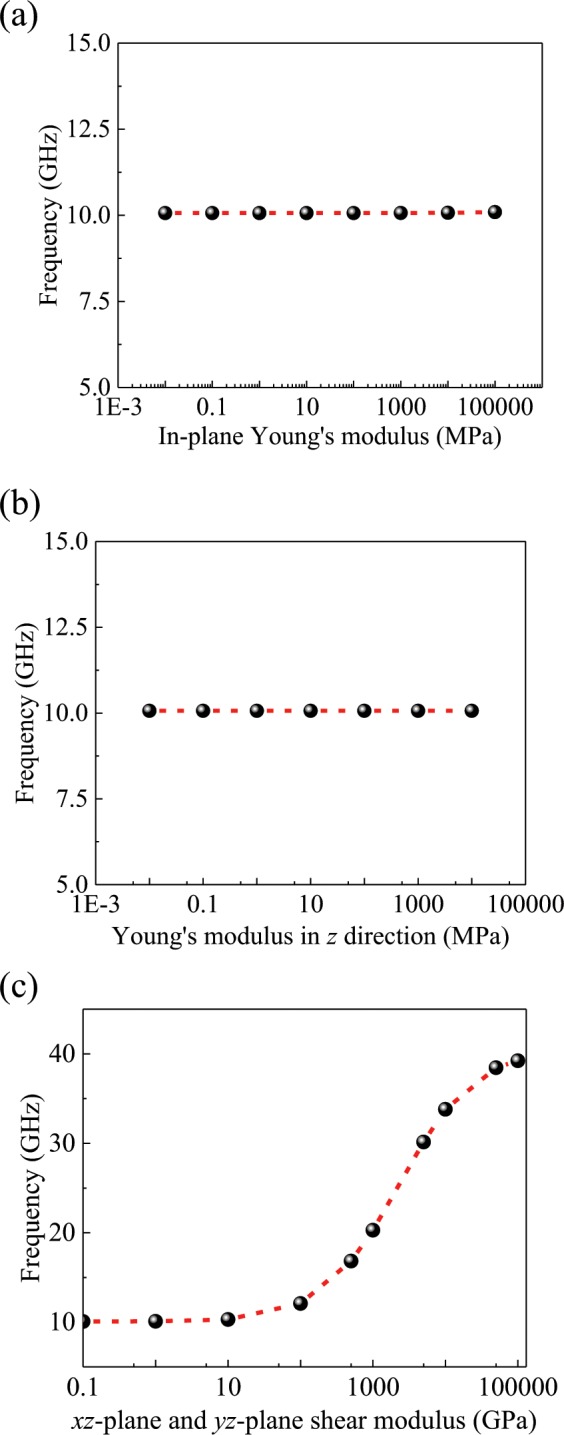
The different factors affect the resonant frequency of the TMLTN. The Young’s modulus of the middle plate in **(a**) the *x*- and *y*-directions and in (**b**) the *z*-direction, and the shear modulus in (**c**) the *xz*- and *yz*-planes, which affect the resonant frequency of the TMLTN.

On the other hand, the SPM is used to investigate the vibration of the TMLTN, as shown in Fig. 3(a). Figure 3(b–d) show the molecular models of the MLBP, MLGS and MLBN, respectively. In Fig. 3(a), the blue layers represent the BP, graphene and BN, and the gray layers represent the one of those three that is caused by the vdW force. Next, the influences of the vdW force on the vibration of the TMLTN are investigated, taking the sandwich plate, which is formed by three plates, as an example, as shown in the inset figure of Fig. 3(a). The mechanical properties of the BP, graphene and BN are given in Table 1^33–35^. Some factors that could affect the resonant frequency of the TMLTN are shown in Fig. 4. Figure 4(a,b) show that the *xy*-plane and the *z*-direction Young’s modulus of the middle plate have slight influence on the resonant frequency of the TMLTN. In Fig. 4(c), when the shear modulus in the *yz*-plane and *xz*-plane of the middle plate continues to increase in the case of the Young’s modulus of the middle plate is being much less than that of the plate representing the BP, graphene or BN, the resonant frequency of the TMLTN increases obviously. Hence, we find that the shear modulus in the *xy*-plane and *xz*-plane caused by the vdW force can affect the resonant frequency of the TMLTN.

**Table 1:** Mechanical properties of BP, graphene and BN.

BP	*D*_11_ (N·m)	*D*_22_ (N·m)	*G*_12_ (GPa)	${{\boldsymbol{\upsilon }}}_{{\bf{12}}}$
	7.9984 × 10^−19^	1.5210 × 10^−19^	52.31	0.054
graphene	*K* = *Eh* (N·m)	${\upsilon }_{2}$		
	251	0.41		
BN	*D*_3_ (N·m)	${\upsilon }_{3}$		
	1.42 × 10^−19^	0.3

**Figure 5:**
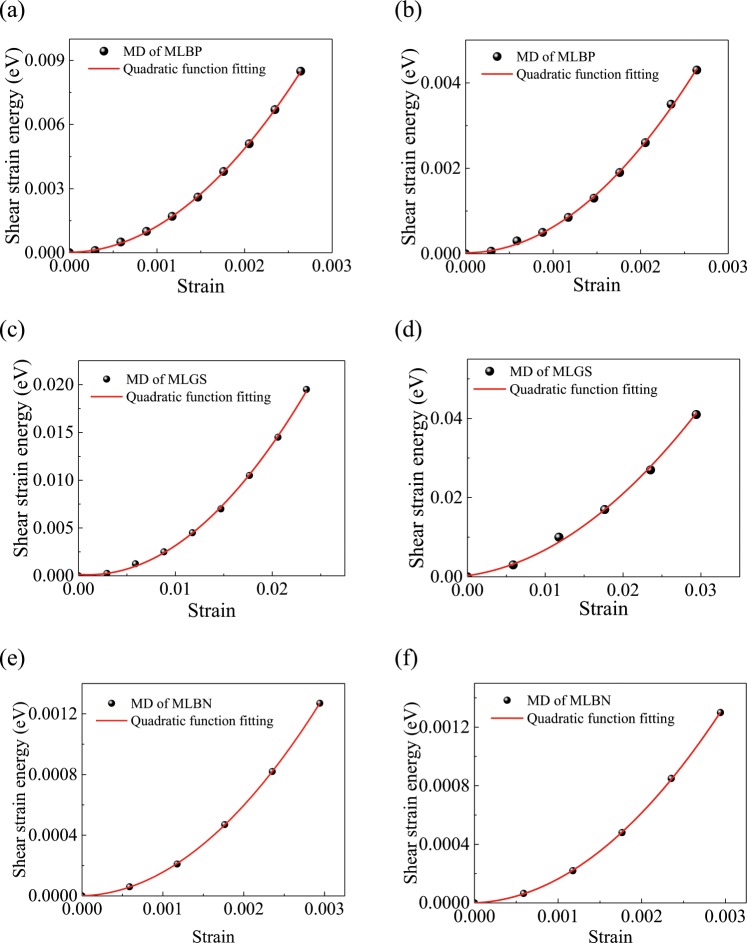
The relationship between the energy of the vdW between two adjacent layers and the strain of the shear strain between two adjacent layers of the TMLTN. The MLBP in the (**a**) *xz*-plane and (**b**) *yz*-plane, the MLGS in the (**c**) *xz*-plane and (**d**) *yz*-plane, the MLBN in the (**e**) *xz*-plane and (**f**) *yz*-plane.

Subsequently, the shear modulus of the *yz*-plane and *xz*-plane between two adjacent layers caused by the vdW force using MD simulations are calculated by

1 $$G=\frac{1}{abh}\frac{{\partial }^{2}U}{\partial {\gamma }^{2}},$$

where *a*, *b* and *h* represent the length, width and distance between two adjacent layers of the TTMLN, respectively. *U* is the energy of the vdW interaction between two adjacent layers. $\gamma$ represents the shear strain between two adjacent layers. In Fig. 5, the relationship between *U* and shear strain $\gamma$ is presented. From Fig. 5 and Eq. (1), the shear modulus caused by the vdW force can be obtained. For the MLBP, the shear moduli in the *xz*- and *yz*-planes are 4.29 GPa and 2.13 GPa, respectively. For the MLGS, the shear moduli in the *xz*- and *yz*-planes are 0.098 GPa and 0.095 GPa, respectively. For the MLBN, the shear moduli in the *xz*- and *yz*-planes are 0.701 GPa and 0.698 GPa, respectively. From the results, it shows that the shear modulus for the MLBP is larger than that for the MLGS or the MLBN. Due to the crystal orientation of the layered material, the shear moduli in the *xz*- and *yz*-planes are different.

**Figure 6:**
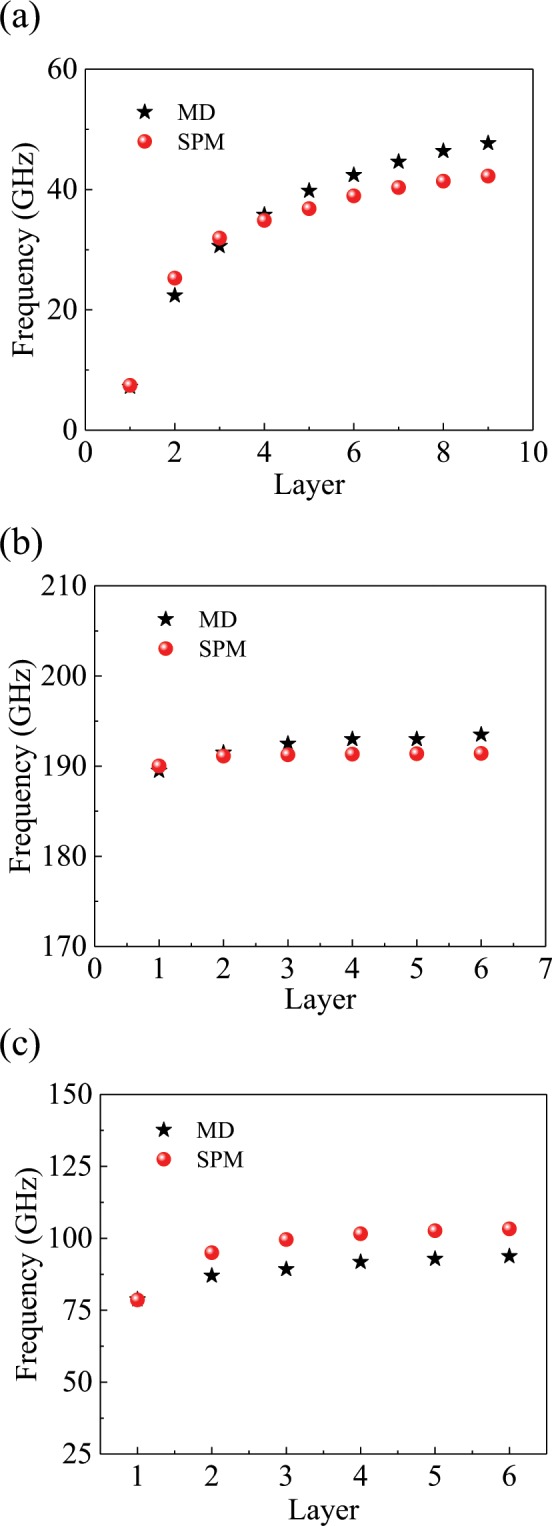
The fundamental resonant frequency of the TMLTN obtained from MD simulations and the SPM. (**a**) MLBP. (**b**) MLGS. (**c**) MLBN.

The vibration of the TMLTN is explored using the SPM, in which the shear modulus caused by the vdW force is considered. The resonant frequency of the sandwich plate is obtained from the finite element method. The fundamental resonant frequency of the MLBP and MLGS obtained from MD simulations and the SPM is presented in Fig. 6. The fundamental resonant frequency obtained by MD simulations is denoted by the red circular points, and the resonant frequency obtained by the SPM is denoted by the black five-pointed stars. The fundamental resonant frequencies of the MLBP, MLGS and MLBN obtained by the SPM are almost the same as those obtained by MD simulations. When the layers of BP and BN increase, the resonant frequency obtained by the SPM also increases. However, when the layers of graphene increase, the resonant frequencies obtained by the SPM have little increase. Hence, the SPM, in which the shear modulus caused by the vdW force is considered, can better predict the vibration of the TMLTN.

The interaction between BN sheets includes electrostatic in nature. The Lennard-Jones potential is used here to model the interaction between layers for the interlayer displacement is small and the structure does not deviate from the relaxed one too much.

## Conclusions

In summary, the MD simulations and the SPM with the shear modulus caused by the vdW force are used to study the vibration of the TMLTN. The resonant frequency increases obviously when the layers of BP and BN increase. However, when the layers of graphene increase, the resonant frequency increases slightly. The shear moduli for MLBP, MLGS and MLBN caused by vdW force are calculated. The results show that the shear modulus for the MLBP is larger than that for the MLGS or the MLBN. Subsequently, it is found that the shear modulus between two adjacent layers caused by the vdW force is the primary reason for this phenomenon. Compared with the MD simulations, the results show that the SPM, in which the shear modulus between two adjacent layers caused by the vdW force is considered, can better forecast the vibration of the TMLTN.

## Methods

### Molecular dynamics simulations

Investigating the dynamic behavior of TTMLNs is employed by MD simulations, which are performed using the LAMMPS package^36^. The Brenner’s second-generation reactive empirical bond order potential^37^, the Stillinger–Weber (SW) potential^38^ and the Tersoff potential^39,40^ are employed to calculate the interactions among in-layer atoms of the graphene, the BP and the BN, respectively. The interactions between the interlayer atoms of TMLTNs are calculated by Lennard-Jones (LJ) potentials, in which the expression for the potential energy is

2 $${{E}}_{{\rm{L}}{\rm{J}}}=4\varepsilon (\frac{{\sigma }^{12}}{{r}^{12}}-\frac{{\sigma }^{6}}{{r}^{6}}),$$

where $\varepsilon$ and $\sigma$ represent the well-depth and the equilibrium distance of the LJ potential, respectively. *r* refers to the distance between interaction atoms. In this paper, the parameters ${\varepsilon }_{{\rm{P}}}=0.0132\,{\rm{eV}}$ and ${\sigma }_{{\rm{P}}}=0.3695\,{\rm{nm}}$ for phosphorus atoms^41^, ${\varepsilon }_{{\rm{C}}}=2.968\,{\rm{m}}{\rm{e}}{\rm{V}}$ and ${\sigma }_{{\rm{C}}}=0.3407\,{\rm{nm}}$ for carbon atoms^42^ are adopted in the MD simulations. The LJ parameters for different types of atoms are obtained from ${\varepsilon }_{{\rm{AB}}}=\sqrt{{\varepsilon }_{{\rm{A}}}{\varepsilon }_{{\rm{B}}}}$ and ${\sigma }_{{\rm{AB}}}=({\sigma }_{{\rm{A}}}+{\sigma }_{{\rm{B}}})/2$. The MD simulations are conducted in a canonical (NVT) ensemble at 100 K with a time step of 1 fs.

### The continuum mechanics method

The SPM is proposed to study the dynamic behavior of TMLTNs considering the interlayer shear effect caused by the vdW interactions. The SPM is shown in Fig. 3(a), the blue layers represent the BP, graphene and BN, and the gray layers represent the one of those three that is caused by the vdW force. The vibration of the SPM is calculated by finite element method.

## Data Availability

All the data will be provided upon reasonable request.

## References

[1] Lee CG, Wei XD, Kysar JW, Hone J (2008). Measurement of the elastic properties and intrinsic strength of monolayer graphene. Science.

[2] Bao Q, Loh KP (2012). Graphene photonics, plasmonics, and broadband optoelectronic devices. ACS Nano.

[3] Bonaccorso F, Sun Z, Hasan T, Ferrari AC (2010). Graphene photonics and optoelectronics. Nat. Photonics.

[4] Ooi KJA, Tan DTH (2017). Nonlinear graphene plasmonics. Proc. R. Soc. A.

[5] Tian H (2014). Wafer-scale integration of graphene-based electronic, optoelectronic and electroacoustic devices. Sci. Rep..

[6] Liang LB (2014). Electronic bandgap and edge reconstruction in phosphorene materials. Nano Lett..

[7] Tran V, Soklaski R, Liang YF, Yang L (2014). Layer-controlled band gap and anisotropic excitons in few-layer black phosphorus. Phys. Rev. B.

[8] Tao J (2015). Mechanical and electrical anisotropy of few-layer black phosphorus. ACS Nano.

[9] Qiao JS, Kong XH, Hu ZX, Yang F, Ji W (2014). High-mobility transport anisotropy and linear dichroism in few-layer black phosphorus. Nat. Commun..

[10] Liu H, Du YC, Deng YX, Ye PD (2015). Semiconducting black phosphorus: synthesis, transport properties and electronic applications. Chem. Soc. Rev..

[11] Topsakal M, Aktürk E, Ciraci S (2009). First-principles study of two- and one-dimensional honeycomb structures of boron nitride. Phys. Rev. B.

[12] Watanabe K, Taniguchi T, Kanda H (2004). Direct-bandgap properties and evidence for ultraviolet lasing of hexagonal boron nitride single crystal. Nature Mater..

[13] Li XB (2015). Structures, stabilities, and electronic properties of defects in monolayer black phosphorus. Sci. Rep..

[14] Liu RM, Wang LF, Zhao JH (2018). Nonlinear vibrations of circular single-layer black phosphorus resonators. Appl. Phys. Lett..

[15] Wang ZH (2015). Black phosphorus nanoelectromechanical resonators vibrating at very high frequencies. Nanoscale.

[16] Mortazavi B, Remond Y (2012). Investigation of tensile response and thermal conductivity of boron-nitride nanosheets using molecular dynamics simulations. Physica E.

[17] Song L (2010). Large scale growth and characterization of atomic hexagonal boron nitride layers. Nano Lett..

[18] Sevik C, Kinaci A, Haskins JB, Cagin T (2011). Characterization of thermal transport in low-dimensional boron nitride nanostructures. Phys. Rev. B.

[19] Wang CR (2016). Superior thermal conductivity in suspended bilayer hexagonal boron nitride. Sci. Rep..

[20] Li BW (2019). Probing van der Waals interactions at two-dimensional heterointerfaces. Nat. Nanotechnol..

[21] Lin K, Zhao YP (2019). Mechanical peeling of van der Waals heterostructures: Theory and simulations. Extreme Mech. Lett..

[22] Zhao YP (2019). Some new mesoscopic crossover length scales concerning the Hamaker constant. Sci. China Tech. Sci..

[23] Xu ZP, Zheng QS (2018). Micro- and nano-mechanics in China: A brief review of recent progress and perspectives. Sci. China: Phys., Mech. Astron..

[24] He XQ, Kitipornchai S, Liew KM (2005). Resonance analysis of multi-layered graphene sheets used as nanoscale resonators. Nanotechnology.

[25] Natsuki T, Shi JX, Ni QQ (2012). Vibration analysis of circular double-layered graphene sheets. J. Appl. Phys..

[26] Liu YL, Xu ZP, Zheng QS (2012). The interlayer shear effect on graphene multilayer resonators. J. Mech. Phys. Solids.

[27] Wang LQ, Kutana A, Zou XL, Ykobson BI (2015). Electro-mechanical anisotropy of phosphorene. Nanoscale.

[28] Zhang HY, Jiang JW (2015). Elastic bending modulus for single-layer black phosphorus. J. Phys. D: Appl. Phys..

[29] Jiang JW (2015). Thermal conduction in single-layer black phosphorus: highly anisotropic. Nanotechnology.

[30] Boldrin L, Scarpa F, Chowdhury R, Adhikari S (2011). Effective mechanical properties of hexagonal boron nitride nanosheets. Nanotechnology.

[31] Klintenberg M (2009). Evolving properties of two-dimensional materials: from graphene to graphite. J. Phys.: Condens. Matter.

[32] Lier GV, Alsenoy CV, Doren VV, Geerlings P (2000). Ab initio study of the elastic properties of single-walled carbon nanotubes and graphene. Chem. Phys. Lett..

[33] Zhang YQ, Wang LF, Jiang JN (2018). Thermal vibration of rectangular single-layered black phosphorus predicted by orthotropic plate model. J. Appl. Phys..

[34] Liu RM, Wang LF (2013). Thermal vibrations of single-layered graphene sheets by molecular dynamics. J. Nanosci. Nanotechno..

[35] Yi JP, Wang LF, Zhang YQ (2018). Vibration of two-dimensional hexagonal boron nitride. Theor. Appl. Mech. Lett..

[36] Plimpton S (1995). Fast parallel algorithms for short-range molecular dynamics. J. Comput. Phys..

[37] Brenner DW (2002). A second-generation reactive empirical bond order (REBO) potential energy expression for hydrocarbons. J. Phys.: Condens. Mat..

[38] Jiang JW (2015). Parametrization of Stillinger–Weber potential based on valence force field model: application to single-layer MoS_2_ and black phosphorus. Nanotechnology.

[39] Sevik C, Kinaci A, Haskins JB, Çağın T (2011). Characterization of thermal transport in low-dimensional boron nitride nanostructures. Phys. Rev. B.

[40] Kinaci A, haskins JB, Sevik C, Çağın T (2012). Thermal conductivity of BN-C nanostructures. Phys. Rev. B.

[41] Rappe AK, Casewit CJ, Colwell KS, Goddard WA, Skiff WM (1992). UFF, a full periodic table force field for molecular mechanics and molecular dynamics simulations. J. Am. Chem. Soc..

[42] He XQ, Kitipornchai S, Liew KM (2005). Buckling analysis of multi-walled carbon nanotubes: a continuum model accounting for van der Waals interaction. J. Mech. Phys. Solids.

